# Placement and Confirmation of Nasogastric Tubes: An Audit of Clinical Practices at a Pakistani Tertiary Care Hospital

**DOI:** 10.7759/cureus.84408

**Published:** 2025-05-19

**Authors:** Aqsa Iqbal, Ali Gohar, Syed Haider Hassan, Saba Waheed, Ammara Saif Ullah, Asim Ali, Maha Saeed, Bilal Ahmed, Haseeb Mehmood Qadri, Asma Nizam

**Affiliations:** 1 Internal Medicine, Lahore General Hospital, Lahore, PAK; 2 Neurological Surgery, Punjab Institute of Neurosciences, Lahore, PAK; 3 Internal Medicine and Medical Education, Azra Naheed Medical College, Lahore, PAK; 4 General Surgery, Lahore General Hospital, Lahore, PAK; 5 Gastroenterology, Lahore General Hospital, Lahore, PAK

**Keywords:** aspiration, clinical audit, intubation, pakistan, patient care, pneumonia, tertiary care centers, x-rays

## Abstract

Background

The widespread and diverse use of nasogastric (NG) tubes healthcare setting and the complications, like aspiration pneumonia that surround its placement call for detailed review of the underlying causes. The shortcomings that lie in the tube placement, securement, maintenance and follow-up are other important factors requiring addressal.

Objective

To evaluate adherence to NG tube placement protocols (confirmation via X-ray/whoosh test) and their impact on aspiration pneumonia rates.

Methodology

We conducted a prospective, clinical audit between June 2024 and July 2024 in the Medical Unit of Lahore General Hospital, Lahore. Patients above 18 years of age with the indication of nasogastric intubation were included. After implementation of the evidence-based protocols, data was collected in three cycles: Cycle 1, Cycle 2 and Cycle 3. Training workshops elaborating the correct tube placement, confirmatory tests and the follow-up care were conducted vigorously at various stages of the cycle to identify whether improvement in patient outcomes occurred or not. Google Forms (Google Inc., Mountain View, CA, USA) and SPSS version 24 (IBM Corp., Armonk, NY, USA) were utilized for data collection and analysis, respectively.

Results

The audit which spanned from 1^st^ June, 2024 till 31^st^ July, 2024 had three cycles of data collection. Each cycle was conducted for 10 days in two months. Pre-implementation phase included 60 patients, followed by 65,80,101 patients in Cycles 1, 2 and 3 respectively. During the first data collection cycle, whoosh test was performed in 50 patients (77%) and chest X-rays were performed on 60% (39 patients) for confirmation. The incidence of aspiration pneumonia dropped from 88.46% (46 patients) in the pre-implementation phase to 54%(n=27) by the end of first cycle. In the second cycle, chest X-rays were performed in 61 (76.25%) NG tube patients, while whoosh test was utilized in 72 patients (90%). The incidence of aspiration pneumonia further decreased, affecting 20.83% of the NG tube patients. By the third cycle, chest X-rays were taken of 96.03% (97 patients) and whoosh test was employed in 101 patients (100%). The incidence of aspiration pneumonia was significantly reduced to 5% among those with NG tube insertion. pH indicator testing was not performed in any patient (0%, 0 patients) in all the cycles of the data collection.

Conclusion

The utilization of chest X-rays in confirming nasogastric tube placement and the staff training and education regarding nasogastric tube maintenance and follow-up care can contribute significantly to lowering the incidence of aspiration pneumonia. Staff training and education regarding the best medical practices for tube insertion, securement and follow-up care is of paramount importance, emphasizing an adoption of a well-rounded approach to reduce the complications related to nasogastric tube placement.

## Introduction

Nasogastric (NG) tubes are indispensable medical tools, particularly for patients with swallowing difficulties or those on ventilators. These tubes provide a vital pathway for nutrition, medication administration, and gastric decompression. However, the effective use of NG tubes hinges on accurate placement and meticulous care to minimize complications and ensure optimal patient outcomes [1].

The gold standard for confirming NG tube placement is a chest X-ray (CXR). However, common practice confirmatory tests include aspirate pH measurement, carbon dioxide detection, and auscultation [2]. Aspiration pH measures gastric acidity, while carbon dioxide detection checks for respiratory tract placement. Auscultation involves listening for gurgling sounds. While these tests provide some indication of placement, they may have limitations and should be used in combination for increased accuracy [3].

Accurate placement of NG tubes is crucial to prevent serious complications, such as aspiration pneumonia, which can lead to inflammation, infection, and respiratory failure [4]. Studies have consistently highlighted the importance of correct placement. A study published in the Journal of Clinical Nursing (2017) found that misplacement rates can be as high as 20% in certain settings, emphasizing the need for standardized protocols and rigorous confirmation techniques [5]. In addition, NG feeding under physical restraint is an intervention that clinicians working in psychiatry units may need to implement [6]. Several factors can influence the accuracy of NG tube placement and the effectiveness of subsequent care. These include staff training and education, standardized protocols, and confirmation techniques [7]. Adequate training and education for healthcare professionals involved in NG tube placement and care are essential to ensure consistent adherence to best practices [8].

This audit aims to evaluate the effectiveness and consistency of NG tube placement and care practices to identify areas for improvement and ensure adherence to best-practice guidelines [9]. By conducting this audit, we seek to identify areas for improvement in NG tube placement and care practices, ensuring that patients receive the highest quality of care and minimizing the risk of associated complications.

## Materials and methods

This prospective clinical audit was carried out at the Department of Medicine at Lahore General Hospital between 1st June 2024 and 31st July 2024. Three cycles of 10 days each were conducted in 60 days. We included patients who were above 18 years of age with indication of nasogastric intubation. We included patients with intact gag reflex, only those who required NG tube placement for medication, gastric decompression, or lavage and patients with altered sensorium having non-intact gag reflex. Patients were excluded only if they did not provide consent or if essential medical records were missing, which would compromise the audit's completeness and data integrity.

Data collection

We used Google Forms (Google Inc., Mountain View, CA, USA) for data collection, which included various details such as demographic information, indications for the procedure, contraindications, NG tube procedure, length and type of the tube, confirmatory tests performed, tube fixation, any complications encountered, the number of attempts, time duration of the NG tube placement, and overall NG tube care.

Prerequisites

The procedure was explained to the patients, and informed consent was obtained. For patients who were unable to give consent themselves, their attendants were informed about the procedure, and consent was subsequently obtained from them. This practice ensures that all patients or their representatives are fully aware of the procedure and its implications, aligning with ethical standards for patient care.

NG tube placement followed hospital protocol involving patient identification, hand hygiene, appropriate positioning (semi-upright), measurement using the nose-ear-xiphoid (NEX) method, lubrication, insertion through the nasal passage, and confirmation of placement using the whoosh test or CXR. All tubes used were standard 14-16 French sizes. A two-day training workshop was conducted before the audit, including one-hour lectures and two-hour practical sessions focusing on tube insertion, securement, confirmation techniques, and complication management. The equipment required for NG tube placement included a clean tray or trolley, disposable gloves, an apron for maintaining hygiene, a fine bore nasogastric tube, an Enteral ISO or Saf syringe (50/60ml), a disposable sick bowl and sterile water. If the patient has a safe swallow and is not nil by mouth, a glass of water with a straw may be provided to help ease the tube insertion. To secure the NG tube in place, an appropriate securing device or hypoallergenic tape is used. Lastly, Conformité Européenne (CE)-marked pH indicator strips are essential for testing human gastric aspirate to confirm proper placement. The NG tubes were inserted by the house officers (Interns) and the postgraduate residents. The nursing staff was responsible for monitoring the tube placement and alerting the doctor in case of tube displacement or spontaneous removal. The NG tube was inserted by the same group of house officers and postgraduate residents and monitored by the same group of nursing staff.

Pre-intervention observation

In every cycle, a different set of patients was recruited. In the first cycle, we observed a total of 60 patients. Out of a total of 60 patients observed, NG tubes were successfully placed in 52 of them. In all 52 cases, the whoosh test was used to confirm the tube placement. However, only 23 patients underwent a CXR to further verify the position of the NG tube, and none of the patients had their tube placement confirmed using a gastric pH indicator. Additionally, 46 patients developed aspiration pneumonia during the observation period. Patients were observed clinically for signs of respiratory distress, fever, cough, or radiographic changes for up to 72 hours post-NG tube insertion. Diagnosis of aspiration pneumonia was based on clinical assessment supported by radiological findings, consistent with a delayed onset typical of aspiration pneumonia rather than aspiration pneumonitis.

Post-intervention assessment

In the second cycle of the audit, we adhered to the guidelines set by the University of Glasgow [10]. To ensure proper implementation, workshops were organized for healthcare professionals and nursing staff, instructing them on how to follow these guidelines effectively. We ensured that a post-NG tube CXR was performed for every patient to confirm proper placement. NG tube care was closely monitored and maintained according to the prescribed guidelines. Following the intervention, a total of 65 patients were observed. NG tube was placed in 50 of these patients. To confirm the correct placement, the whoosh test was performed in all of them. CXR was conducted in 39 patients. CXR, although considered the gold standard, was not performed routinely for all patients due to limitations in resource availability, the high patient load in the emergency and medical wards, and the need for prompt initiation of feeding or medication. X-ray confirmation was reserved for cases where bedside confirmation was inconclusive or where complications were suspected. The whoosh test for NGT placement involves rapidly injecting air through the tube while listening with a stethoscope over the epigastrium (upper abdomen).

A "whooshing" sound indicates the air is entering the stomach, suggesting correct placement. Absence of a sound suggests the tube tip might be elsewhere (lung, esophagus, etc.). The gastric pH indicator test was not conducted in any of the patients. 

Multiple workshops, two per week, were conducted to enhance the implementation of Glasgow guidelines, and a third cycle was necessary for cycle completion due to the inconclusive results of the first two cycles. In the third cycle of the audit, NG was placed in a total of 101 patients. A whoosh test was performed for all of them, while CXRs were conducted in 97 patients to verify placement. Workshops were conducted after each audit cycle to present findings in the form of presentations, highlight deviations from best practices, and provide hands-on training for staff on correct NG tube placement and confirmation methods. These interactive sessions aimed to improve compliance and awareness through practical demonstrations and discussions.

## Results

The pre-implementation data included 60 patients, of whom 52 had NG tubes in place. Among these 52 patients, all underwent the whoosh test to confirm tube placement, while only 23 patients had CXRs, and none underwent pH indicator testing. A significant 76.6% (46 out of 60) of the total patients developed aspiration pneumonia, and 88.46% of the NG tube patients were affected.

During the first data recollection cycle, the sample size increased to 65 patients, with 50 patients having NG tubes. All 50 NG tube patients underwent the whoosh test, and CXRs were performed on 39 (60%) of them for confirmation. Similar to the pre-implementation phase, no patients underwent pH indicator testing. The incidence of aspiration pneumonia dropped to 41% (27 out of 65) in total, with 54% of the NG tube patients developing the condition.

In the second cycle of data collection, 80 patients were included, with 72 patients having NG tubes. As in previous cycles, all 72 patients with NG tubes had the whoosh test, while CXRs were performed for 61 (76.25%) of them. The use of pH indicator tests remained absent. The incidence of aspiration pneumonia further decreased to 18.75% (15 out of 80) in total, affecting 20.83% of the NG tube patients.

By the third data recollection cycle, the study population grew to 101 patients, all of whom had NG tubes. Every patient with an NG tube underwent the whoosh test, and 97 (96.03%) patients also had CXRs. However, the pH indicator test was still not utilized. The incidence of aspiration pneumonia was significantly reduced to 5% (five out of 101) among both the total patient population and those with NG tube insertion. The summary of results is illustrated in Table 1.

**Table 1:** The progressive reduction in aspiration pneumonia rates across the cycles NG: nasogastric

Cycle	Total Patients	NG Tube Patients	Whoosh Test	Chest X-ray	pH Indicator Test	Aspiration Pneumonia (Total Patients)	Aspiration Pneumonia (NG Tube Patients)
Pre-Implementation	60	52	52	23	0	46 (76.6%)	46 (88.46%)
Cycle 1	65	50	50	39	0	27 (41%)	27 (54%)
Cycle 2	80	72	72	61	0	15 (18.75%)	15 (20.83%)
Cycle 3	101	101	101	97	0	5 (5%)	5 (5%)

## Discussion

NG tubes are widely used in hospitalized patients as a temporary method for delivering nutrition, hydration and medications. Given their significance, NG tubes must be inserted, positioned, and secured accurately, adhering to guidelines to prevent critical complications such as aspiration, which can be fatal. A nasogastric tube is considered to be correctly placed when its tip is positioned anywhere from the mid-stomach to the first portion of the jejunum [11]. While there are various methods to confirm the placement of NG tubes, pH testing is often considered the initial choice for both adults and infants. Other methods include checking the color of aspirate, auscultation, carbon dioxide testing, ultrasound, and testing for bilirubin, pepsin, or trypsin. However, chest and abdominal X-rays remain the most reliable way to ensure accurate tube placement [12].

In our study, following the guidelines of the University of Glasgow, we used the whoosh test and CXRs as the main methods to confirm the position of the NG tube [10]. We observed a significant reduction in the incidence of aspiration pneumonia across multiple cycles of data collection. The progressive decline in aspiration pneumonia rates - from 88.46 % in the pre-implementation phase to just 5% in the final cycle - highlights the impact of improved adherence to clinical guidelines and practices surrounding NG tube management.

One of the critical factors contributing to this positive outcome was the increased use of CXRs for the confirmation of NG tube placement. In the pre-implementation phase, only 23 out of 52 patients with NG tubes underwent this essential imaging, leading to a high rate of aspiration pneumonia. The findings of this audit raise concerns about the reliability of the whoosh test in confirming NG tube placement, especially given the high rates of aspiration pneumonia observed in the early audit cycles. Recent studies highlight that the whoosh test alone is not sufficiently accurate and may lead to false assurance of correct placement. It lacks sensitivity and specificity, and therefore, radiographic or pH-based confirmation is recommended whenever feasible [12,13]. However, as the data collection cycles progressed, the number of patients receiving CXRs increased, culminating in 97 out of 101 patients in the final cycle being evaluated. The use of this gold standard method in confirming the proper positioning of NG tubes is likely the major factor involved in reducing aspiration pneumonia rates. While X-rays are considered the gold standard method to confirm NG tube placement, they have several drawbacks. These include the risk of misinterpretation, delays in feeding, exposure to radiation, and high costs [13]. In our audit, all patients who underwent CXR confirmation were found to have correctly positioned NG tubes. Aspiration pneumonia occurred only in patients whose tube placement was confirmed by the whoosh test alone. Notably, all patients who underwent X-ray had also received the whoosh test beforehand. Additionally, it's not always practical to repeatedly order X-rays to confirm NG tube placement to start enteral feeding, especially in critical care settings where patients frequently change positions between supine and prone [14]. With healthcare professionals more experienced in recognizing the signs of tube misplacement and responding appropriately, it stands to reason that the incidence of related complications would decrease. This shift emphasizes the importance of staffing and training in alleviating risks associated with NG tube feeding.

Although the pH indicator test is common, cost-effective, and considered a first-line method for verifying NG tube placement, it was not utilized throughout the study due to the unavailability of pH testing equipment and budgetary constraints. Factors such as the inability to obtain an aspirate or the use of medications like proton pump inhibitors or antacids that can interfere with pH testing make CXRs necessary to ensure accurate tube placement [15]. While the Nasogastric podcast (NGPod) system, a novel fiberoptic pH test device, offers a promising alternative to reduce the need for repeated aspirates and CXRs [16], its availability and cost are also limitations in our healthcare system. This limitation raises important questions about the adequacy of resources for ensuring optimal patient care.

It is inevitable to highlight that different hospitals follow slightly different protocols depending on the circumstances. Being a high-flow tertiary care center, there is a shortage of nursing staff which would explain the shift in the responsibility of managing NG tubes to house officers and post-graduate residents. A cross-sectional, prospective audit conducted in the Department of Surgery of the same hospital in 2022 illustrates that consents for emergency surgery were being obtained by house officers and rarely by post-graduate residents [17]. Another quality improvement project was conducted in the Department of Radiology, Jinnah Hospital, Lahore, about the reporting of soft tissue masses on ultrasonography which highlights the importance of improving the ongoing current practices, targeting relevant healthcare professionals [18]. While efforts are being made to train the house officers and residents on structured reporting guidelines, the effectiveness may vary from individual to individual, and according to the situation in resource-limited settings.

Limitation

There are potential limitations to this study. The sample size, while increasing over time, may still be relatively small for definitive conclusions. Additionally, the study did not investigate other potential risk factors for aspiration pneumonia, such as patient characteristics and co-morbidities. pH indicator testing was not performed due to equipment unavailability and restricting comparison with CXRs. Heavy reliance on CXRs increases costs and radiation exposure, limiting practical use in all settings. Inconsistent adherence to guidelines in earlier cycles affected data accuracy. The absence of advanced tools like the NGPOD system limited the use of safer alternatives. Training impact was not measured for long-term effectiveness in NG tube management. 

Recommendations

Considering the validity of chest and abdominal X-rays in confirming correct NG tube placement, it vastly helps if these show the entire length of the tube in particular showing where the NG tube tip and ports are located. When the NG tube's correct placement has been confirmed by X-ray, it also helps to make a marking at the exit site, either at the nares or the lips. Healthcare professionals must follow this to ensure correct tube assessment. In patients with altered levels of consciousness or who are currently delirious or confused, utilization of X-rays can be imperative considering a higher risk of having an undetected displaced tube. As there is a possibility of NG tube dislodgement, these must be checked by clinicians at least once daily and also before giving feed or medicines.

## Conclusions

Improved nasogastric tube management can reduce the incidence of aspiration pneumonia and the overall patient morbidity and mortality. A more thorough study with a greater patient population is needed for drawing exact inferences, but the improvement in patient care outcomes with enhanced nasogastric tube management protocols does point in a positive direction. Though the use of pH indicator testing is the first line for confirming nasogastric tube placement, the limitation lies in the equipment availability, but with the presence of effective adjuncts like CXRs, nasogastric tube placement can still be confirmed with reasonable accuracy in a resource-limited setting. Also, staff training and education regarding the best medical practices for tube insertion, securement and follow-up care is of paramount importance, emphasizing an adoption of a well-rounded approach if the complications related to nasogastric tube placement have to be drastically reduced. By conducting this audit, the areas of inaccuracy of techniques were explored and there is a need to conduct regular audits according to standard guidelines. While the audit revealed a potential association between protocol adherence and reduced aspiration pneumonia rates, causality cannot be established due to the observational design. Further interventional studies are recommended to confirm these findings.
